# A Case of a Neuroendocrine Tumor in a Liver Transplant Patient: Diagnostic and Management Difficulties

**DOI:** 10.3390/life15030421

**Published:** 2025-03-07

**Authors:** Carmen Colaci, Caterina Mercuri, Alessandro Corea, Rocco Spagnuolo, Patrizia Doldo

**Affiliations:** 1 Department of Health Sciences, University “Magna Graecia”, 88100 Catanzaro, Italy; spagnuolo@unicz.it; 2 Department of Clinical and Experimental Medicine, University “Magna Graecia”, 88100 Catanzaro, Italy; c.mercuri@unicz.it (C.M.); alessandro.coales@gmail.com (A.C.); doldo@unicz.it (P.D.)

**Keywords:** NET, liver, immunosuppressive therapy, liver transplantation, HCC, holangiocarcinoma

## Abstract

Neuroendocrine tumors (NETs) of the liver are a rare entity. NETs are often poorly recognized, with diagnostic difficulties and differential challenges between primary tumors of the liver and metastases from other organs, mainly from the gastrointestinal tract. Multidisciplinary and multi-technical diagnosis is mandatory to properly treat these tumors. This case describes the complex history and the treatment course of a 68-year-old man with a history of NET onset after liver transplantation. Liver transplantation is the treatment of choice for patients with advanced liver disease or acute liver failure, but careful pre- and post-transplant patient monitoring is required. Liver transplant patients receive immunosuppressive therapy, and donor livers should be screened to exclude potential malignancies. This clinical case, in addition to emphasizing the diagnostic and therapeutic difficulty of hepatic NET, underlines the role of post-transplant immunosuppressive therapy and pre-transplant screening, which includes a thorough evaluation of donor and recipient history, physical examination, and laboratory tests. Moreover, post-transplant immunosuppressive therapy is essential to maintain the viability of the transplanted organ, but it is not free from potential risks, including an increased risk of cancer. Therefore, close monitoring of therapy is necessary to optimize long-term results and the patient’s quality of life.

## 1. Introduction

Neuroendocrine tumors arise from neuro-ectodermal cells, which are dispersed throughout the body. They are characterized by slow growth and the ability to secrete hormones or biogenic amines [1]. They often originate from the gastroenteropancreatic (GEP) tract (GEP-NET) and the bronchopulmonary tree. In addition, they present variable behavior and prognosis. In fact, their aggressiveness depends on the site of origin. For instance, NETs of the small intestine have a high malignant potential, whereas gastric and rectal NETs have a low tendency to metastasize but can progress rapidly once they become metastatic [2]. Despite being rare entities, the incidence and prevalence of NETs continue to increase globally, with the highest rates in the United States, Canada, and Norway [3]. Due to the indolent nature of the disease, its prevalence represents an imminent health crisis. It may come as a surprise that gastrointestinal NETs are now the second most prevalent gastrointestinal malignancy after colon cancer. The current prevalence of NETs in the United States amounts to approximately 170,000 patients [4]. GEP NETs are the most common subtype, accounting for 55–70% of all NETs. In comparison, primary hepatic neuroendocrine tumors (PHNETs) are extremely rare neoplasms, with only 94 documented cases as of 2009, first identified by Edmondson in 1958. Due to their rarity, it is crucial to exclude metastatic NETs and primary liver tumors, such as hepatocellular carcinoma (HCC) and cholangiocarcinoma (CCA), before diagnosing PHNETs [5]. A key role in the occurrence of post-liver transplant tumors could be played by immunosuppressive therapy [6]. The presented report is the first case in the literature of de novo PHNET on a liver transplant and is designed to focus on the diagnostic and therapeutic difficulties of managing the transplanted liver and on the importance of carefully monitoring the patient before and after the transplant.

## 2. Case Presentation

Here, we report the case of a 68-year-old male admitted to our Gastroenterology Unit in January 2024 after the report of a lesion of uncertain significance in the principal biliary duct.

Our patient was a former smoker and previous alcohol drinker, allergic to ceftriaxone. He had no family history of neoplastic diseases of the gastrointestinal tract and was hypertensive on furosemide treatment. In 2019, he underwent orthotopic liver transplantation for HCC, which occurred on alcoholic cirrhosis, and has been on immunosuppressive therapy with tacrolimus 3 mg 1 tablet daily since then. At the end of June 2023, following the appearance of hyperchromic urine and acholic stools, the patient underwent routine blood examinations with findings of increased liver indices of cytolysis and cholestasis. Therefore, in July 2023, he underwent magnetic resonance cholangiography (MR cholangiography) at another clinical center, which showed a stenosis of the main bile duct with dilatation of the intra- and extra-hepatic bile ducts and a lesion of approximately 40 mm surrounding the bile duct, portal vein, and common hepatic artery. On 14 August 2023, the patient arrived at the emergency room of the former hospital for generalized itching and abdominal pain. Blood tests confirmed increases in the cytolysis and cholestasis indices: total bilirubin—11.4 mg/dL (n.v. 0.3–1 mg/dL), AST—91 U/L (n.v. 5–40 U/L), ALT—138 U/L (n.v. 5–35 U/L), and GGT—495 U/L (n.v. 11–60 U/L). There were also increases in the tumor markers: carcinoembryonal antigen (CEA)—9.3 ng/mL (n.v. 0–4ng/mL), alpha fetal protein (AFP)—12.15 ng/mL (n.v. 0–7 ng/mL), and Ca—19.9 at 4000 U/mL (n.v. 0–37 U/mL).

**Figure 1:**
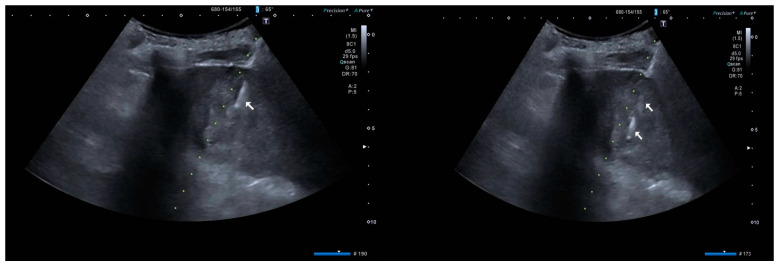
Ultrasound image of biopsied liver segment VI lesion. The arrow indicates the presence of the VI liver segment.

An abdominal CT scan with contrast was also performed at admission, which confirmed a marked dilatation of the intrahepatic bile ducts in both hemispheres, recognizable up to a few mm downstream of the hepatic hilum, and the presence of hypodense tissue of suspected malignant significance surrounding the main bile duct (which was no longer recognizable), the portal vein, the proper hepatic artery, and the distal portion of the common hepatic artery, measuring 40 mm, similar to the previous MR cholangiography. No other liver nodules or abdominal organ changes were observed, nor were intraperitoneal or retroperitoneal fluid collections/effusions. In ordered to perform biopsies to determine the nature of the lesion found on the abdominal CT scan, the patient underwent percutaneous transhepatic cholangiography, which confirmed marked dilatation of the intrahepatic bile ducts and obstruction of the common hepatic duct, without contrast medium flow downstream. Due to the inability to pass through the obstruction, biopsies could not be taken. Therefore, an external biliary 8F caliber drain and a pig-tail tip placed at the level of the obstruction was inserted. Subsequently, 4 days later, the patient underwent a total-body positron emission tomography (PET) scan, which showed the presence of a hepatic hilar finding in the same area as the tissue component seen in the previous CT scan, characterized by high glycolytic metabolism of suspected heteroplastic nature. No other suspected heteroplastic lesions were found during the PET scan. On 4 September 2023, the patient underwent exploratory laparotomy with subsequent biliary–digestive anastomosis. Histological examination of the surgical specimen showed, at the level of the vegetating tissue of the biliary tract, biliary mucosa with papillary architecture and high-grade dysplasia. Meanwhile, in the common hepatic duct tract, fragments of biliary wall showed extensive fibrosclerosis and mild chronic phlogosis, limited by diathermocoagulative artefacts. On 29 September 2023, the patient had his first oncological visit to our hospital for suspected biliary cancer, given the strong radiological and laboratory evidence, despite the histological examination of the biopsies taken during the exploratory laparotomy being conclusive. Another abdominal MRI at our center confirmed the presence of a 40 mm formation, at the hepatic hilum, of heterogeneous nature, infiltrating the portal vein, bile ducts, and part of the common bile duct. Additionally, this lesion appeared to not be divisible from the head of the pancreas. A new repetitive 4 cm lesion was also identified in the VI hepatic segment. The presence of peritoneal fluid and signs of portal hypertension with splenomegaly were reported. In January 2024, the patient was admitted to our gastroenterology unit and underwent an ultrasound-guided liver biopsy of the VI hepatic segment lesion (Figure 1).

**Figure 2:**
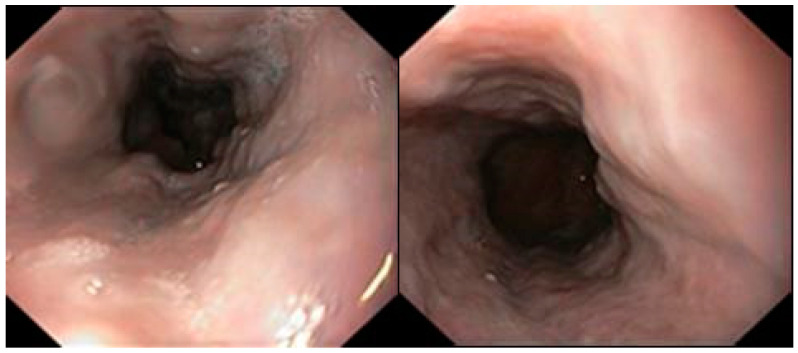
F1 esophageal varices in gastroscopy: the **left** image shows the portion of the middle esophagus; the **right** image shows the distal esophagus.

**Figure 3:**
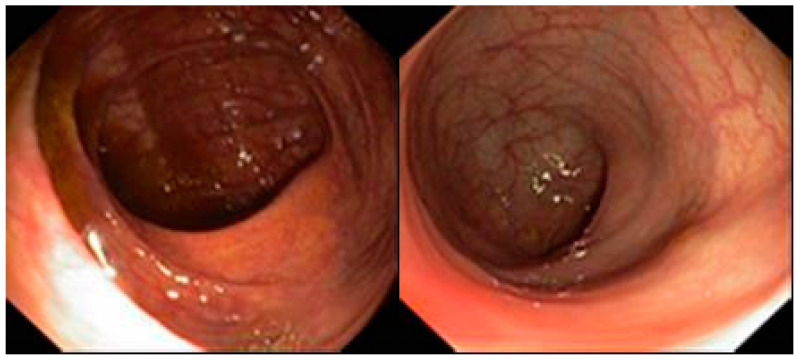
Normal colonoscopy findings: the **left** image shows the cecum; the **right** image shows the descending colon.

The histological examination of the biopsy showed the presence of poorly differentiated neuroendocrine carcinoma (NEC), whose immunophenotype was reported as CK 19+, CK7−, CK20−, CDX2+, synaptophysin+, Ki67 90%. During hospitalization, the patient underwent colonoscopy and gastroscopy, and F1 esophageal varices (Japan Research Society of Portal Hypertension—JRSPH) were shown (Figure 2). No other pathological findings were shown (Figure 3).

Then, systemic therapy with carboplatin and etoposide was initiated. On 15 February 2024, the patient started the first cycle of therapy, completed without any side effects. Further disease reassessment with a total body CT scan was postponed due to the patient’s strong desire to return home. On 2 April 2024, the patient was scheduled for the second cycle of therapy, but due to Grade 1 thrombocytopenia, the treatment session was postponed. Since then, the patient has not received any systemic therapy due to persistent thrombocytopenia. On 3 June 2024, a follow-up total body CT scan confirmed the presence of the known hepatic hilar formation and the 4 cm repetitive lesion in the VI hepatic segment, but also multiple irregular hypodense formations with peripheral contrast enhancement in the right hepatic sections, suspected for nodules of HCC. The treatment of choice for the newly evidenced liver lesions was a combination of bevacizumab and atezolizumab, but due to the patient’s history of liver transplantation and immunosuppressive therapy, no treatment was feasible at that time.

## 3. Discussion

Liver transplantation is the preferred treatment for patients with advanced liver disease or acute liver failure. From 1968 to 2018, a total of 80,000 transplants were performed worldwide. In 2023 alone, Italy conducted 39 liver transplants. It is estimated that survival rates have significantly improved over the last 25 years, reaching 96% and 71% at 1 and 10 years post-transplant, respectively [1]. Several observational studies have shown that the rate of de novo neoplasms in liver transplant patients increases 2-fold for malignant neoplasms of solid organs and 30-fold for lymphoproliferative neoplasms compared to the general population [2,3]. However, the rate of lymphoproliferative neoplasms in liver transplant recipients is lower than in recipients of other solid organ transplants, and the incidence of these neoplasms develops in the first 12–18 months post-transplant [7]. New malignancies of solid organs occur most commonly after the first year post-transplant and are associated with increasing age of the recipient. Liver transplant patients who have a history of alcoholic liver disease or primary sclerosing cholangitis present a higher risk [8]. The most frequent neoplasms include non-melanoma skin cancer and esophageal and colorectal neoplasms [8]. No data are available in the literature about NET development in liver transplant patients. The occurrence of PHNET is extremely rare in a liver transplant patient. Currently, our clinical case is the only one reported in the literature. Numerous risk factors are associated with the occurrence of NETs. Since they are rare entities, it is difficult to obtain clear data. Family history of malignancy, cigarette smoking, and alcohol consumption have been observed to increase the risk of developing NETs. A further relationship exists between liver diseases, such as cirrhosis and hepatitis, and the development of NETs. Such conditions can alter the normal metabolism of hormones and neurotransmitters, which can influence the growth of NETs. For example, cirrhosis can lead to increased levels of certain growth factors and cytokines that can promote tumor growth. In addition, liver dysfunction can impair the liver’s ability to metabolize hormones produced by NETs, causing systemic effects and exacerbating symptoms [9]. Generally, NETs are asymptomatic, except for the eventual excessive production of certain hormones, which, depending on the substance produced, can cause diarrhea and facial flushing (carcinoid syndrome), hypoglycemia (insulinoma), heartburn, and vomiting (gastrinoma) [2]. PHNETs present unique clinical features, characterized by slow and often asymptomatic growth until advanced stages. They are often detected incidentally and may present as endocrinologically silent liver masses [10]. Notably, only 6.8% of PHNET cases are associated with the classic carcinoid syndrome related to liver metastases from extrahepatic NETs. The diagnosis is commonly made because of the mass effect in the liver or adjacent structures, which may lead to symptoms such as abdominal distension, non-specific pain, jaundice, and a palpable mass in the right upper quadrant [11]. In our case, the patient had no carcinoid syndrome but mainly cholestasis symptoms, such as generalized itching and abdominal pain with the appearance of acholic stools and hyperchromic urine. Diagnosing PHNETs is complex and often involves misdiagnosis as hepatocellular carcinoma (HCC) or cholangiocarcinoma (CCA) due to overlapping imaging features. CCA, while rare, is more common than PHNETs and presents various symptoms based on its type [2]. Over the past three decades, the incidence of CCA has reportedly increased, with higher rates observed in the United States and among Hispanic and Asian populations compared to African-American populations [11]. CCA is classified into three types based on the location of tumor development: intrahepatic CCA (iCCA), perihilar CCA (pCCA), and distal CCA (dCCA), corresponding to the intrahepatic bile duct, right and left bile ducts, common hepatic duct, and cystic duct, respectively. Patients with pCCA and dCCA typically present with jaundice and elevated cholestasis markers, while those with iCCA often exhibit non-specific symptoms [12]. In fact, given the increased indexes of cholestasis and the MRI imaging of a formation occupying the hepatic hilum, infiltrating the portal vein, the biliary tract, and part of the principal biliary duct, our first diagnostic hypothesis was CCA. For these reasons, in the context of our case, the initial diagnostic hypothesis was more than acceptable. The gross radiological features of PHNETs can be very varied. They can present as solid or cystic lesions, as well as diffuse or well-circumscribed in margins. In addition, as shown in the study of Wang et al., they are richly vascularized by the hepatic artery [13]. However, HCC and CCA also exhibit moderate vascularity, with HCC characterized by marked arterial enhancement and delayed washout during portal phases, while CCA progressively absorbs contrast during arterial and venous phases. Despite advances in imaging techniques, the risk of diagnostic errors and the challenges in obtaining a correct differential diagnosis remain high [14]. The diagnostic evaluation of PHNETs often requires additional examinations, such as PET scans, gastroscopy, colonoscopy, bronchoscopy, and octreotide scans [15]. In our patient, gastroscopy and colonoscopy were negative. Instead, the total body PET scan confirmed the hepatic hilar finding with features of high glycolytic metabolism.

Needle biopsies can help in the preoperative diagnosis of PHNETs, but their accuracy is often insufficient. Consequently, the diagnosis can only be confirmed after surgery [16]. Furthermore, immunohistochemical markers, such as neuron-specific enolase, chromogranin A, and synaptophysin, are used for their high sensitivity in the diagnosis of neuroendocrine tumors [17]. In our case, needle biopsies were sufficient for diagnosis. There are no guidelines for the treatment of PHNET, and surgical resection is the option of choice. Patients with PHNETs that are not candidates for surgery may undergo trans-arterial chemo embolization (TACE), which provides tumor mass reduction and symptoms control, but the current evidence is still limited [18]. The interesting point of this case is the occurrence de novo of the NET on a transplanted liver. The role of immunosuppressive therapy is essential for successful liver transplantation but carries a risk of toxicity. This therapy increases the likelihood of developing cancers, such as non-melanoma skin cancer, hepatocellular carcinoma (HCC) recurrence, non-Hodgkin lymphoma, and lung and renal cell cancers [19]. Calcineurin inhibitors are associated with a higher neoplastic risk, while mTOR inhibitors, particularly sirolimus, may reduce the risk of HCC recurrence and non-melanoma skin cancer. However, there are no data on the effects of mTOR inhibitors on other malignancies [20]. Because of the above, patients who are candidates for liver transplantation must be screened for the presence of any malignancy [21]. In addition, donors should also be carefully selected. In this regard, the literature reports a case of a PHNET in a patient who received a liver transplant from a donor. Immediately after the liver transplantation for alcoholic cirrhosis, a hypoechogenic lesion (15 mm) in the left hepatic lobe was found on abdominal ultrasonography, while liver biopsy confirmed it to be a PHNET [22]. Genetic analysis of the PHNET confirmed the donor origin of the neoplasm, although the donor had been screened for malignancies [23]. For our case, the transplant was performed in 2019, and since then, the patient has been receiving immunosuppressive treatment with tacrolimus. A genetic investigation was not performed to rule out the possibility that the new lesion could have originated from the donor liver. It seems unlikely that the PHNET is donor-derived, as no suspicious lesions had emerged in imaging checks prior to August 2023. There is no evidence regarding the development of PHNET in patients on immunosuppressive therapy with tacrolimus. On the contrary, sirolimus, an agent belonging to the mTOR inhibitors, such as tacrolimus, is now studied for the treatment of lung, pancreatic, and gastrointestinal NET [22].

## 4. Conclusions

This case report highlights the diagnostic and therapeutic challenges of PHNET in patients already undergoing liver transplantation. PHNET remains a poorly understood condition that must undergo differential diagnosis from metastatic NETs in other organs and major liver malignancies, such as HCC and cholangiocarcinoma. Moreover, the fact that it is a clinically silent lesion makes its detection mostly incidental. Despite their rarity, the incidence of these tumors is increasing, probably due to improved diagnostic techniques and increased awareness among healthcare professionals. It is crucial that healthcare professionals are vigilant in monitoring transplanted patients for the presence of these lesions to ensure timely and appropriate treatment. Overall, a personalized approach to the management of PHNET is crucial to improve the outcome and quality of life of these patients. Pre-transplant screening is a key element in optimizing outcomes and reducing complications. After transplantation, a further crucial role is played by immunosuppressive therapy in preventing organ rejection, but the latter must be carefully managed due to the risk of associated infectious and neoplastic complications. Research into the molecular mechanisms underlying neuroendocrine tumors of the liver is ongoing, with the hope of developing more effective therapeutic options.

## Data Availability

No new data were created or analyzed in this study.
